# Predicting Veterinary Career Intentions Using Motivational Characteristics: A Survey Study Among Hungarian Students

**DOI:** 10.3390/vetsci12121189

**Published:** 2025-12-12

**Authors:** Laura Szücs, Péter Fehérvári, László Ózsvári

**Affiliations:** 1 Department of Veterinary Forensics and Economics, University of Veterinary Medicine Budapest, István Street 2, 1078 Budapest, Hungary; szucs.laura.hu@gmail.com; 2 Department of Biostatistics, University of Veterinary Medicine Budapest, István Street 2, 1078 Budapest, Hungary; fehervari.peter@univet.hu

**Keywords:** veterinary career intentions, motivational characteristics, high school students, childhood animal exposure, CART analysis, veterinary education, career guidance

## Abstract

The path to becoming a veterinarian often begins well before university education, so understanding students’ career choices is essential. This study aimed to identify motivational characteristics of Hungarian high school students interested in veterinary medicine. Between December 2022 and March 2023, a questionnaire was distributed during high school career days, university open days, and via online platforms to collect data on students’ backgrounds, motivations, childhood animal exposure, and alternative career options. Recursive conditional Classification and Regression Tree (CART) models were used to identify motivational characteristics predicting veterinary career intentions. Among 428 respondents (74.1% female; mean age 17.8 years), a fondness for animals emerged as the predominant motivational factor; 97.4% had childhood pets, most commonly dogs. Human medicine was the main alternative career, followed by agriculture and veterinary nursing. Most students were interested in small animal medicine, while horse-related experience strongly predicted interest in equine practice. Interest in agriculture predicted preference for farm animal care. Students inclined toward non-clinical roles showed stronger interest in natural sciences and decided on a veterinary career later in life. These findings suggest that many students commit to veterinary medicine before age 12, highlighting the need for early engagement through competitions, camps, and extracurricular activities.

## Simple Summary

Understanding why young people choose to become veterinarians can help universities improve both recruitment and training. In this study, we surveyed Hungarian high school students who were considering applying to the University of Veterinary Medicine Budapest. We explored their motivations, childhood experiences with animals, and alternative career options. Most students had long-standing exposure to animals and developed an interest in veterinary medicine early in life, often before the age of 12. A strong fondness for animals was the main motivation, and many students preferred small animal practice. However, experience with horses or interest in agriculture strongly predicted interest in equine or farm animal medicine, respectively. These findings highlight the importance of early outreach programs, such as animal-focused competitions, camps, and extracurricular activities, to support future veterinary students.

## 1. Introduction

In most countries, a veterinary degree is a prerequisite for professional registration; thus, the university admissions process plays a pivotal role in shaping the future composition of the profession. Veterinary schools, therefore, act as critical gatekeepers, determining who gains entry into the field and ultimately influencing the direction in which the profession evolves [1]. Based on our experience, the majority of Hungarian veterinary students intend to pursue clinical careers, particularly in small animal practice, from the outset of their studies, and they are generally not easily dissuaded from this path. By supporting students in reaching the level required to begin veterinary studies, and by admitting individuals from diverse backgrounds, we may increase the likelihood of recruiting future veterinarians into less popular fields.

Veterinary medicine is a continuously evolving profession, and the pace of change shows no signs of slowing. In recent decades, the composition of the veterinary workforce has shifted significantly, driven by advancements in agricultural technology, a steady rise in pet ownership, and an increasing number of women entering the profession [2,3,4,5]. As a result, new career paths have emerged, and the role of veterinarians in society has also undergone transformation [2,6]. From treating individual diseased farm animals, our roles shifted to an advisory role in the livestock sector and to a very different role in small animal practices. There, the veterinarians’ role is more akin to a human doctor’s than the veterinarians of the last century. Despite these changes, access to veterinary education remains largely limited to students from privileged socio-economic backgrounds [7,8]. At the same time, interest in livestock- and rural-based veterinary careers is steadily declining [3,9], presenting new and urgent challenges for the profession. A growing concern of the veterinary community is that traditional educational models and professional role expectations may no longer align with the demands of modern veterinary practice. Becoming a successful veterinarian today may require a different skill set and mindset than in the past. As expectations surrounding the veterinary role—as well as teaching and learning methods—continue to evolve on a global scale, there is a clear need for the veterinary universities to reassess and adapt current training practices to better prepare future professionals for these emerging realities.

In general, choosing a profession—particularly one as academically demanding as veterinary medicine—is a decision typically made at an early age, usually before high school [1,7,8,10,11,12,13]. This is largely due to the importance of receiving the appropriate high school education to qualify for university admission. As a result, students who remain uncertain about their career goals early in life may find it increasingly difficult to pursue competitive high school programs later. Ideally, all high school students would have access to structured career guidance systems to support informed decision-making. However, such systems are often underdeveloped or inconsistently implemented. As a consequence, some students may choose career paths based on wrong motives or inadequate information, and may not be fully aware of the harsh realities of the profession they are entering [14]. This disconnect can lead to increased dropout rates, early career exits, and the exclusion of students who might be better suited for the profession [14].

It is reasonable to speak of a predisposition toward both healthcare and agricultural professions, including veterinary medicine. This aptitude is shaped by a combination of personality traits, interests, self-confidence, and both internal and external motivational characteristics, goals and expectations [11,12,15,16,17,18,19,20]. When these expectations are misaligned, engagement becomes superficial, and aspirations are more likely to be abandoned [15,16,18]. Moreover, a strong element of “self-selection” is present in the higher education application process. Students who perceive themselves as unlikely to succeed may choose not to apply at all. In evaluating their chances, students consider not only their academic achievements, but also their socio-economic background and perceived opportunities [21,22,23]. This dynamic is expected to be present in Hungary as well, especially given that access to quality high school education may be limited for individuals from lower- to middle-class backgrounds.

In most countries, including Hungary, the veterinary profession has traditionally been male-dominated. However, this trend began to reverse in 2004, when—for the first time—more women than men graduated as veterinary surgeons in Hungary. This marked the beginning of an accelerated gender shift, which led to the proportion of male veterinary graduates falling below 25% between 2011 and 2016 [5]. This rapid gender shift has contributed to labour shortages in certain areas of veterinary medicine, particularly in large animal practice, where working conditions are demanding, working hours are long, and achieving work–life balance can be especially challenging, particularly for women [5]. Moreover, a previous study from Hungary showed that both farmers and colleagues in the region are biased towards male veterinarians in livestock practices, making the labour shortages even more pronounced given the current gender distribution [5]. However, blaming labour shortages in large animal veterinary practices only on the feminization of the profession would be short-sighted. Living and working in a rural area often has its own challenges, such as lower return for the hours worked, more on-call shifts, a lack of infrastructure (such as schools or hospitals) and fewer opportunities for leisure activities [24,25].

When designing the admission process for veterinary programs, it is essential to assess not only applicants’ academic qualifications but also their suitability for the profession [1]. This way, we may reduce the likelihood of individuals leaving the profession due to initially mistaken assumptions. To do this effectively, we must gain insight into the motivational characteristics and intrinsic goals that drive students to pursue veterinary medicine [26,27]. Understanding these variables may also help predict outcomes such as mental well-being, risk of burnout, and the likelihood of attrition from the profession [28,29].

The primary aim of this study was to gain a deeper understanding of the motivational characteristics predicting high school students’ decision to pursue veterinary medicine. We used Self-Determination Theory (SDT) as a basis to design our study. We examined intrinsic and extrinsic motivational characteristics and goals among aspiring veterinary students and hypothesized that socio-demographic background plays a significant role in shaping their core motivations [15,16,18,30]. Identifying and understanding these underlying drivers can support the development of more targeted educational interventions that may play a key role in helping students successfully navigate the university admission process and pursue veterinary studies with greater confidence and preparedness. Building on the findings of this study, we intend to carry out additional research to support the development of targeted programs that can be implemented in Central and Eastern Europe.

## 2. Materials and Methods

### 2.1. Study Design

The survey was drafted to evaluate the background of students interested in veterinary medicine, their main motivational characteristics, their pets and other career options. The drafted survey was reviewed by undergraduate veterinary students (*n* = 5), Ph.D. students (*n* = 2) and academic professionals (*n* = 3) to receive feedback on its content. Based on collected feedback, revisions were made before the survey was sent to potential respondents. This study collected and analysed quantitative data. The structured questionnaire was divided into two main sections (Supplementary Material).

The first section focused on the high school respondents’ socio-demographic background, type of high school education attended, and their evaluation of biology and chemistry education at their schools. These subjects were emphasized due to their relevance in the veterinary admissions process, where students are typically required to take advanced-level exams in both disciplines.

The second section explored motivational characteristics, asking students to assess the degree to which specific variables predicted their decision to pursue veterinary studies. These characteristics are based on the Self-Determination Theory, although we adapted them to better suit the context of our study. Responses were recorded on a 5-point Likert scale, where 1 indicated “*Did not influence me at all*” and 5 indicated “*Had a strong influence on me*”. We chose this scaling method because the Hungarian grading system also uses a 1 to 5 scale, which students are most familiar with. The intrinsic motivational characteristics included: “*I would like to work with animals*”, “*I would like to cure animals*”, “*I have always wanted to become a vet*”, “*I like animals*”, “*I am interested in agriculture*”, “*I nursed a sick animal back to health*”, “*I can always learn something new*”. Extrinsic motivational characteristics included: “*A family member or friend is a veterinarian*”. “*It is easy to find a job*”, “*It is a respected profession*”, “*The salary is good*”, “*Veterinarians are needed all around the world*”. Additionally, the questionnaire surveyed childhood pet ownership, as early experiences with animals were assumed to be influential in shaping long-term interests. Students were also requested to indicate the species they were most interested in treating in the future, as well as the age at which they decided to become veterinarians and any alternative careers they had considered. Further questions explored students’ job shadowing experience, based on the assumption that those with firsthand exposure to veterinary practice often possess a clearer understanding of the profession. Finally, students were invited to report their awareness of scholarships, programs, and competitions for high school students, as well as advanced-level preparatory courses offered by the University of Veterinary Medicine Budapest (UVMB). The questionnaire also included items assessing demand for additional programs to support veterinary career exploration and informed decision-making.

### 2.2. Data Collection and Processing

The questionnaire was made available in both online format via Google Forms (Google LLC, Mountain View, CA, USA) and as a printed hard copy, and data were collected between December 2022 and March 2023. Distribution took place during high school career days, job fairs, higher education expos, and by email to students who had registered for the Open Day at UVMB. During the Open Day, prospective students visited the campus to gain firsthand insight into university life. The event aimed to provide detailed information about the admission process, curriculum, and student experience, and to address any questions participants had regarding veterinary education at UVMB. In addition, the questionnaire was shared through targeted Facebook groups related to biology and chemistry education and university preparatory courses. This included a dedicated group for students enrolled in UVMB’s preparatory program. Participation in the survey was voluntary and anonymous. Prior to completing the questionnaire, each participant was required to provide written informed consent. All responses were coded to detect potential data entry errors or inconsistencies. The collected data were processed using Microsoft Excel 2019 (Microsoft Corp., Redmond, WA, USA). As part of this process, we transformed Yes/No answers to 1/0, as this numerical format was considered easier to handle.

### 2.3. Statistical Analysis

A total of 496 responses were collected, of which 68 were incomplete and excluded from the analysis (13.7%). This left 428 valid questionnaires for further processing. After a detailed descriptive analysis of all measured variables, we applied Classification and Regression Tree (CART) models using the *party* package in R (version 4.1.2; R Foundation for Statistical Computing, Vienna, Austria) [31] to explore relationships between students’ motivational characteristics and relevant background factors.

CART, a tree-based machine learning method, was chosen for its ability to model complex, potentially non-linear relationships without requiring stringent parametric assumptions. Specifically, we implemented conditional inference trees using the ctree() function from the *party* package [31]. This algorithm selects variables and split points based on statistical inference using permutation tests, thereby avoiding the selection bias typical of traditional CART algorithms such as rpart [31].

Our primary outcome variable was Career Intent, defined as a binary indicator of whether a student expressed a strong intention to pursue a veterinary career (1 = high intent, 0 = low intent). Predictor variables included demographic factors (e.g., gender, urban vs. rural background), academic background (e.g., high school specialization), and motivational characteristics measured via Likert scales (1–5; see above), such as passion for animals, desire for financial security, and interest in research. These Likert-scale measures were later integrated as predictor variables into the CART models.

No predictors were eliminated prior to analysis; all collected variables with potential relevance to career choice were retained. Categorical predictors were coded as factors, and continuous variables were standardized where appropriate to enhance interpretability of the resulting splits. The tree-growing process was limited to a maximum depth of 3, so the models are not fragmented, and statistical significance at each node was determined using permutation-based tests with a threshold of *p* = 0.05. The R code can be found in the Supplementary Material.

Although conditional inference trees can handle missing values via surrogate splits, the main analysis was conducted on a complete-case dataset (*N* = 428). A supplementary analysis including cases with missing values confirmed that the results were not substantively altered. The classification performance of the final tree was also quantified. Using the full analytic dataset, the overall classification accuracy was 78.3% (misclassification rate: 21.7%), with a balanced accuracy of 72.2%. A 50-fold cross-validation procedure yielded a mean accuracy of 75.5% (SD = 4.2%).

## 3. Results

### 3.1. Demographics and Education

Among high school students considering a career in veterinary medicine, the majority (74.1%) identify as female, and 25.9% as male. The mean age of respondents was 17.8 years, with a median age of 17 (±SD = 3.5). The youngest participants were 12-year-old female students, while the oldest was a 54-year-old male dentist. Regarding place of residence, the largest proportion of respondents lived in cities (36.7%), followed by townships/villages (23.6%), Budapest, the capital city (20.6%), and county seats (19.2%). This distribution shows that less than one-quarter of students interested in veterinary medicine came from truly rural areas.

In terms of educational background, most respondents attended general high schools (72.4%) or specialized high schools (22.2%), while only a small proportion attended technical schools (5.4%). Specialized high schools were more common among female students and those from Budapest and its surrounding areas, while technical schools were more commonly attended by male students and those from rural regions. Students were invited to rate the quality of biology and chemistry education they received at their respective schools. Biology education was rated higher than chemistry overall (mean scores: 3.97 vs. 3.57). Students from Budapest and the central region did not report higher scores than those from rural areas (Biology: 3.96 vs. 3.98; Chemistry: 3.58 vs. 3.53). However, subject ratings increased with the degree of school specialization. Students from specialized schools rated their biology education substantially higher (4.30) than those from general high schools (3.94) or technical schools (3.04). A similar trend was observed in chemistry (4.11 vs. 3.49 vs. 2.35). Gender differences were also present: female students reported receiving slightly better education in both subjects than their male peers (Biology: 3.99 vs. 3.93; Chemistry: 3.66 vs. 3.50) (Table 1).

### 3.2. Motivational Characteristics

**Figure 1:**
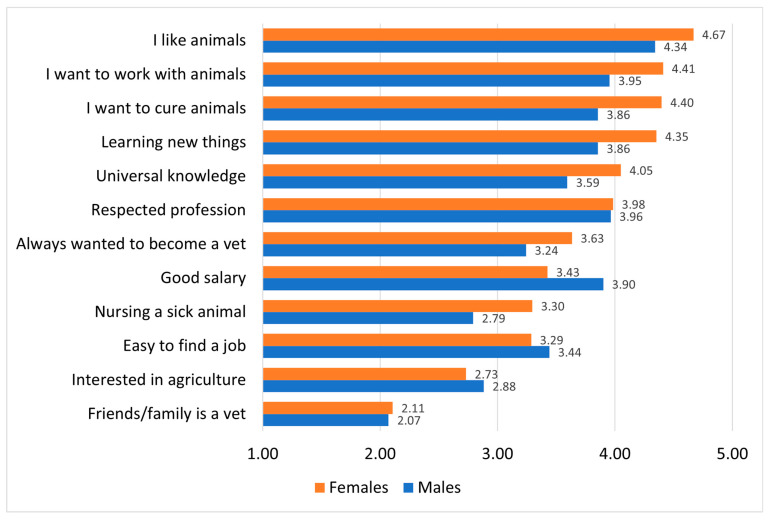
Motivational characteristics influencing veterinary career choice by gender. Note: Likert scale from 1 to 5, where 1 means “Not at all” and 5 means “Very strongly”.

Intrinsic motivational characteristics were generally more influential for female students, except for “*I am interested in agriculture*,” where male students scored slightly higher. In contrast, extrinsic motivational characteristics showed less pronounced gender differences. Connections with veterinarians and the perceived prestige of the profession were equally influential for both genders, while “*Easy to find a job*” and “*Good salary*” were rated as more important by males. Among all motivational factors, “*I like animals*” emerged as the most influential, while “*Friends or family members are veterinarians*” was the least influential for both genders (Figure 1).

Differences in motivation were also observed based on place of residence, particularly in terms of human–animal relationships. Students from rural areas—especially from townships—tended to report that the love of animals and their interest in agriculture shaped their career choice more strongly. Prestige and high income were also more frequently cited as important by these students. In contrast, high school students from larger cities more often described becoming veterinarians as a long-standing childhood dream (Table 2). Most of them (89.7%) stated that they “*definitely want to become a veterinarian*” as well.

**Figure 2:**
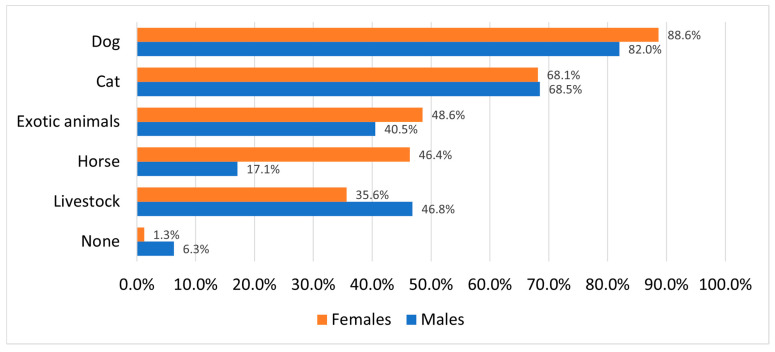
Childhood animal exposure by gender.

We considered pet ownership to be a potentially relevant characteristic. Only 2.6% of our respondents reported having no animals in the household during childhood. The vast majority grew up with dogs (87.0%) and cats (68.2%), but exotic animals—such as birds, rabbits, fish, and small rodents—were also relatively common (31%). Equestrian experience showed a strong gender disparity: 46.4% of female respondents had experience with horses during childhood, compared to just 17.1% of males. In contrast to equestrian experience, exposure to livestock was more closely associated with place of residence. Overall, 38.7% of high school students reported growing up with horses, while 36.8% had direct contact with livestock animals during their childhood (Figure 2).

### 3.3. Common Alternative Career Choices

**Figure 3:**
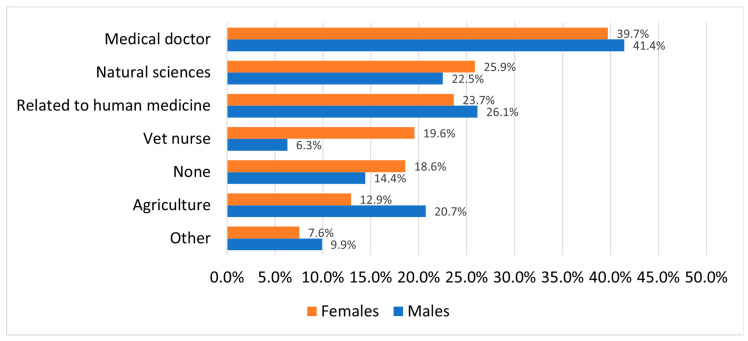
Common alternative career choices by gender.

Female students appear to be slightly more committed to pursuing veterinary medicine, with 18.6% reporting that they do not consider any alternative careers, compared to only 14.4% of male students. The most cited alternative to veterinary medicine was human medicine: approximately 40% of students stated that they were also considering becoming a medical doctor. Additionally, 24.5% expressed interest in other healthcare-related professions such as dentistry, paramedicine, nursing, or physiotherapy. Careers in the natural sciences—particularly biology or zoology—were also frequently mentioned, accounting for 19.8% of responses. Another prominent backup plan was becoming a veterinary nurse (16.2%), a path that was more commonly considered by female students. Given the close relationship between veterinary medicine and agriculture, it is not surprising that 15.3% of respondents considered careers in agricultural fields. Notably, male students and those from townships were almost three times more likely to express interest in agriculture-related careers compared to other groups. A smaller proportion of respondents (8.2%) reported considering unrelated professions such as law, law enforcement, or similar fields (Figure 3).

### 3.4. Veterinary Field Preferences

**Figure 4:**
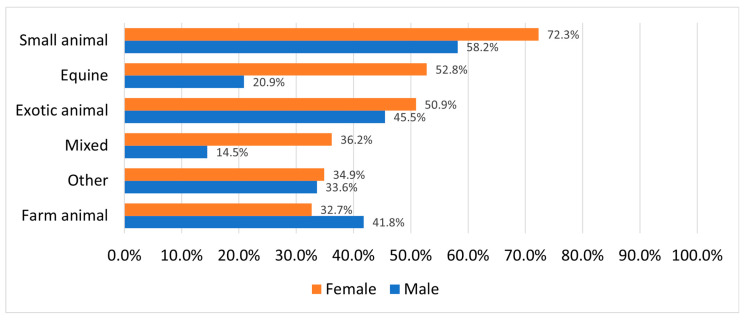
Veterinary field preferences by gender.

For most respondents, the small animal practice was the preferred professional field; however, it was much more common amongst females and somewhat more common in respondents residing in cities. Among those students who had horses growing up, small animal practice came second after equine practice. Females were more interested in treating horses than males; amongst females, equine practice was the second most common choice, while amongst males, it was only the fifth. If a student had horses, they were very likely to be interested in treating them (83.1%) as opposed to those who only had dogs and/or cats (9.9%). Farm animal practice was more common amongst males than females (second choice vs. fourth choice) and somewhat more common amongst students living in the countryside. If a student only had dogs and/or cats, they were very unlikely to be interested in treating farm animals (the least common choice). Exotic practice was slightly more attractive to females and to those who lived in bigger towns. If a student had any type of exotic animal, the chances of them being interested in treating exotic species considerably increased. A student was considered to be interested in mixed animal practice if they were interested in treating horses and/or livestock in addition to small animals and/or exotic animals. We found that in the whole sample, the level of interest in mixed animal practice was very similar to that for equine practice. Laboratory work, education and state veterinary practice were the least common options in most socio-demographic groups. If a student only had dogs and/or cats they were more likely to be interested in lab work (22.5%) and education (21.1%) than in any other field including large animal fields, namely equine practice (9.9%), state veterinary practice (2.82%) or farm animal practice (1.4%) (Figure 4, Table 3).

### 3.5. Motivational Characteristics of Veterinary Field Preferences (CART)

**Figure 5:**
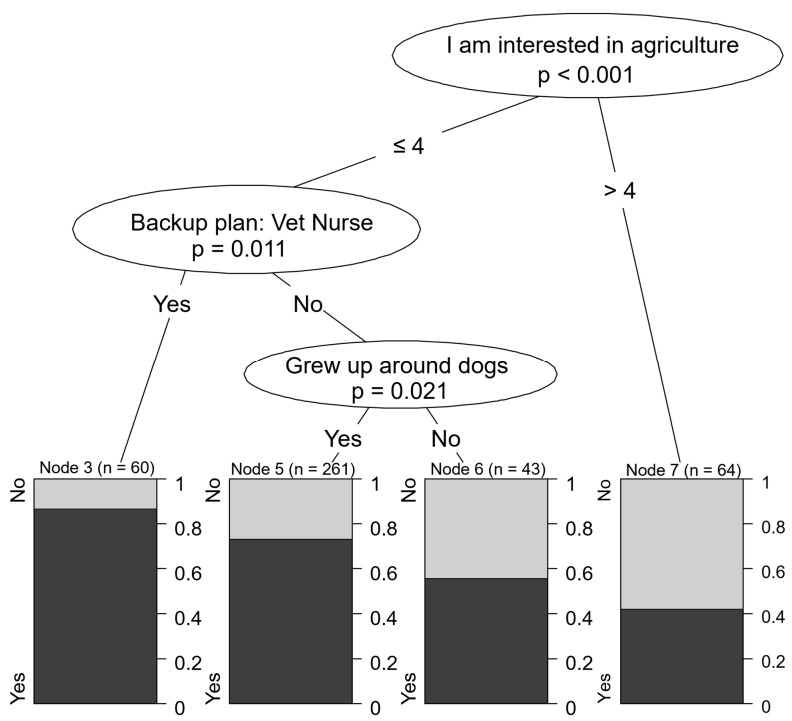
Motivational characteristics associated with preference for small animal practice.

The most common career choice amongst students interested in veterinary medicine was small animal practice (68.7%). High school students who were not interested in agriculture or did not consider veterinary nursing as a backup plan showed the highest level of interest in small animal practice (84.7% of all respondents). Even if they did not consider becoming a veterinary nurse, students who owned a dog (76.9%) were still more inclined toward small animal medicine compared to those without a dog. Nevertheless, a large portion of the latter group expressed interest in treating small animals, which was the most popular area of interest across most sociodemographic groups (41.0% of all respondents) (Figure 5).

**Figure 6:**
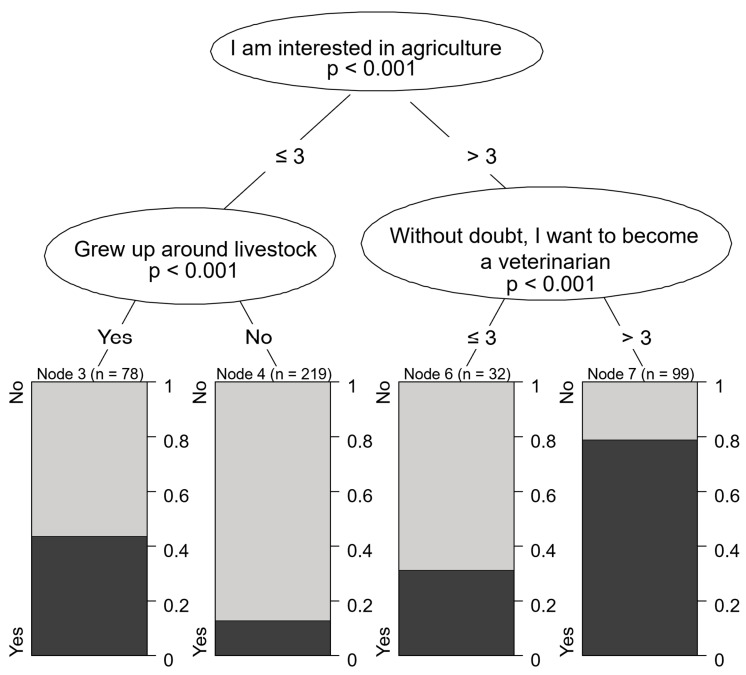
Motivational characteristics associated with preference for farm animal practice.

The main motivational characteristics of students aspiring to enter food animal practice were a focus of attention at UVMB in recent years, as this is the field facing the most severe labor shortages. Students who expressed an interest in agriculture and reported high motivation were more likely to be interested in treating farm animals than those with lower levels of motivation (79.8% vs. lower proportions in other groups). High school students who were less interested in agriculture but had some experience with livestock growing up were also more likely to be drawn to farm animal practice (42.4%) compared to those without such experience (16.1%) (Figure 6).

**Figure 7:**
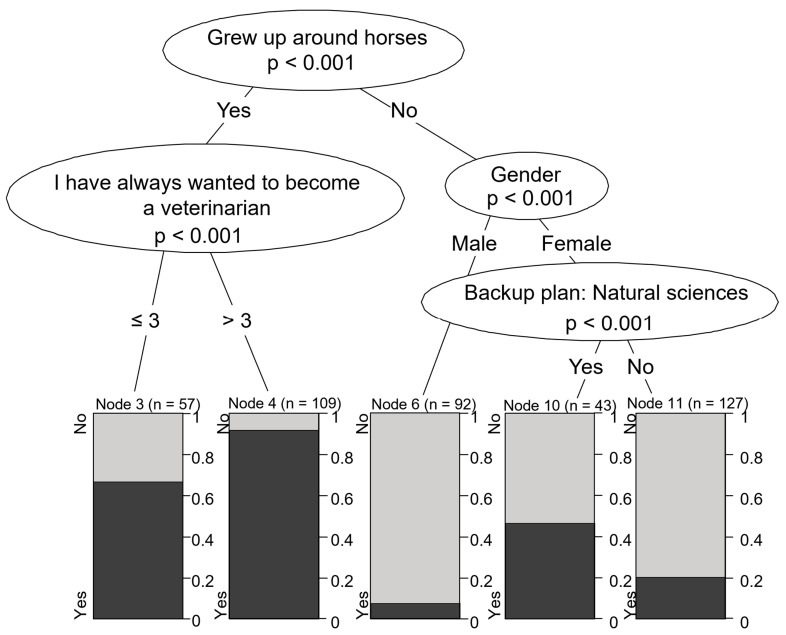
Motivational characteristics associated with preference for equine practice.

**Figure 8:**
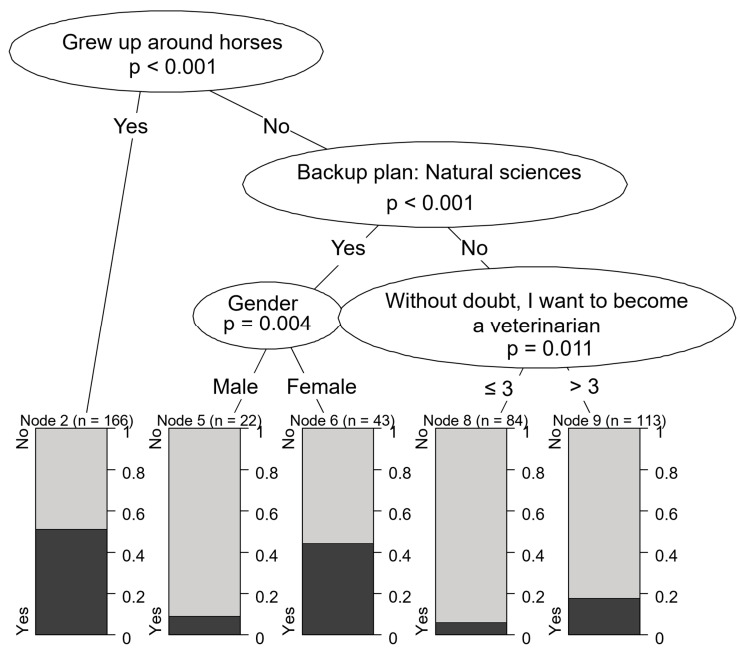
Motivational characteristics associated with preference for mixed animal practice.

Interest in equine practice was strongly associated with horse ownership in the family household. Students whose family owned a horse (or frequently interacted with horses growing up) and had long aspired to become veterinarians were the most likely to be interested in equine practice (96.2%). However, even those whose family owned a horse but did not always plan to become a vet were still very likely to be interested in treating horses (64.1%). Conversely, males without interaction with horses during childhood were highly unlikely to be drawn to equine practice (8.4%). Females without childhood experience with horses but who were interested in other scientific careers, such as biology or zoology, showed a slightly higher likelihood of pursuing equine practice (42.1%) compared to those not considering these careers (20.2%) (Figure 7). Motivations for equine and mixed practices were quite similar (Figure 8).

**Figure 9:**
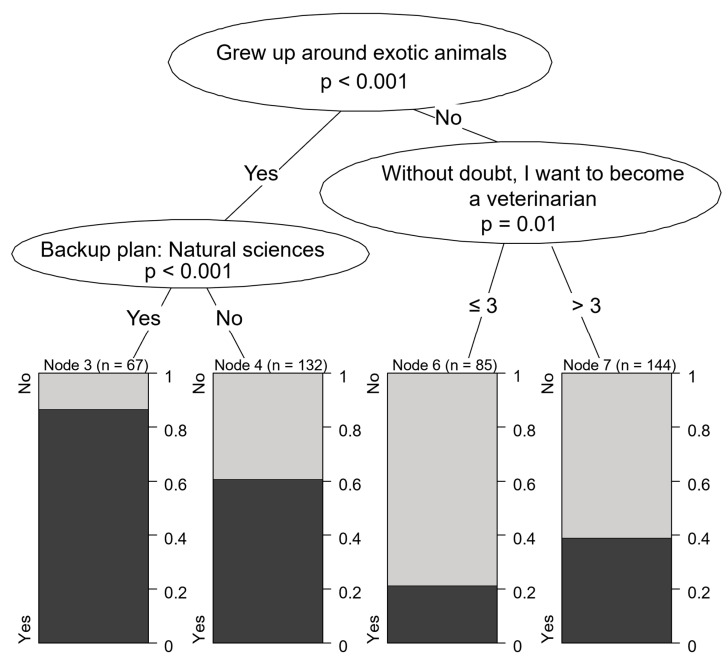
Motivational characteristics associated with preference for exotic animal practice.

Exotic animal practice primarily attracted students who had some type of exotic pet growing up, particularly those with an interest in other natural sciences (86.2%). Students without childhood experience with exotic pets were somewhat more likely to be interested in treating them if they were highly motivated toward the veterinary profession (39.7% vs. 21.0%) (Figure 9).

**Figure 10:**
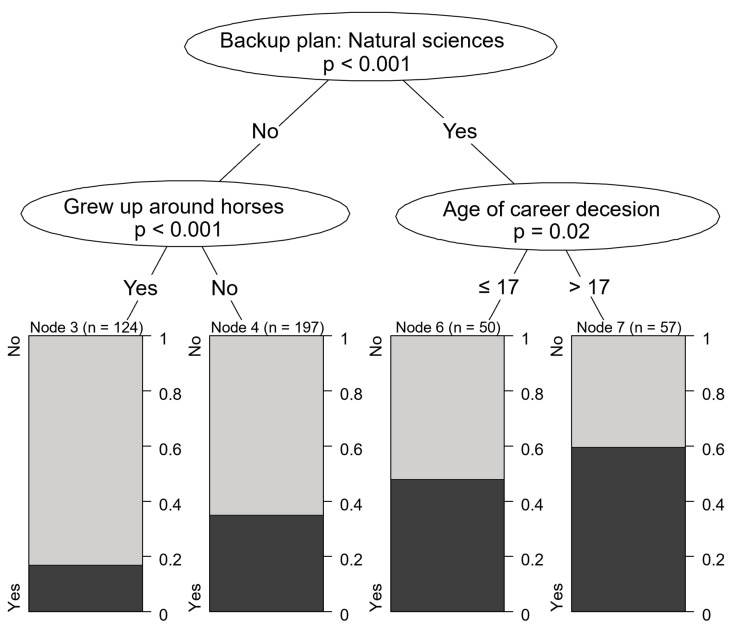
Motivational characteristics associated with preference for non-clinical veterinary fields.

The surveyed high school students demonstrated the greatest openness to non-clinical branches of veterinary medicine (e.g., laboratory work, veterinary public health, or academic roles) when they also considered other science-related career paths. This tendency was more common among those who chose to pursue veterinary studies later in life (60.0% vs. 48.1%). Among students uninterested in the natural sciences, those without prior experience with horses were particularly unlikely to consider non-clinical veterinary careers (18.2%), instead showing a preference for equine or mixed animal practice. Even students from households without a horse tend to show less interest in non-clinical roles (Figure 10).

**Figure 11:**
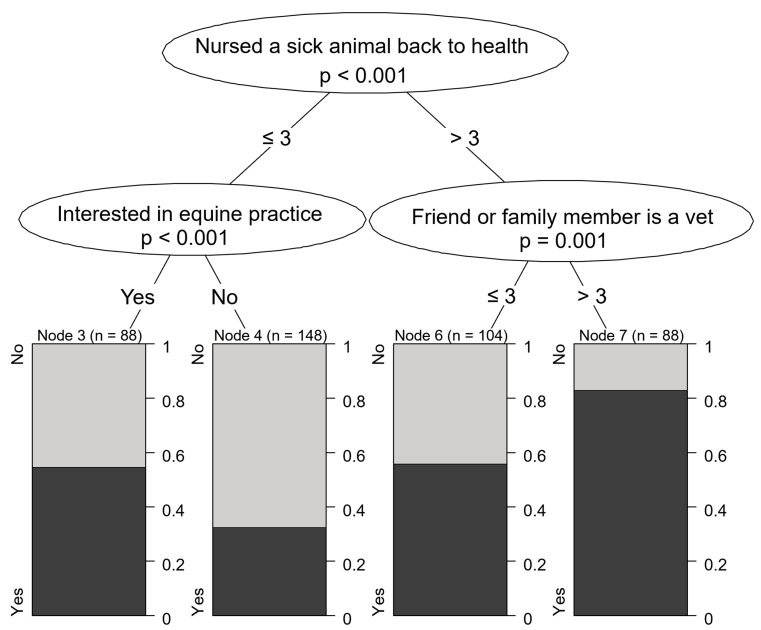
Motivational characteristics associated with job shadowing.

Conversely, students who had previously cared for a sick animal were more likely to have participated in job shadowing, and this likelihood further increased if they had a veterinarian—whether a family member or a friend—whom they could observe (82.1%) (Figure 11). Interestingly, even those who were less influenced by the experience of nursing an animal back to health, but whose family owned a horse, still showed a high likelihood of having completed job shadowing (57.0%).

## 4. Discussion

### 4.1. Socio-Demographic Characteristics of Hungarian Veterinary Entrants and UVMB Admissions

Comparing our current findings with a previous Hungarian study [21], the demographic composition of the Hungarian veterinary student population of UVMB has remained largely unchanged since 2016. The gender ratio continues to be skewed, with approximately 70–75% female and 25–30% male students. Around half of all veterinary students still come from the capital and the surrounding region, while only a small proportion originate from smaller townships. Notably, just 20–23% of first-year students are from rural settlements outside cities or towns. Given that students with a background in livestock farming are more likely to pursue professional fields related to food animals, this trend raises concerns—especially considering that most Hungarian veterinarians working in these fields today are middle-aged or older men [5], and that the age, gender, and field preferences of Hungarian veterinarians largely mirror those observed in other European countries [32].

In Hungary, all students are theoretically eligible for free higher education if they meet the academic criteria: successful completion of the Matura exam in the required subjects by the university (alongside the mandatory subjects) and achieving a required composite score—based on academic performance—than the threshold determined by the number of applicants versus available places. However, these requirements may appear unattainable to students who did not attend specialized secondary schools. While all high school students are expected to receive equal-quality education, in practice, this is typically accessible only in specialized high schools located in central regions or major university cities. Students from rural areas, particularly those who attended technical schools—most often males involved with agriculture and livestock (as our current data indicates)—face lower chances of being admitted to veterinary programs through the standard route.

One of the inspirations for our project was the “Gateway to the Profession” program at the Royal Veterinary College (RVC), which serves as a promising example of how to bridge the gap between privileged and underprivileged yet highly motivated students. Through intensive tutoring and practical experience during a preparatory “year zero”, all participants in the program successfully passed their exams and stood out for their commitment and resilience during their subsequent academic years [7,8,22]. The UVMB is similarly committed to supporting students from disadvantaged backgrounds. We offer additional scholarships to those interested in production animal medicine, food chain safety, and public veterinary services. Moreover, during the admissions process, we aim to identify key motivational characteristics among applicants and reward those who demonstrate strong vocational commitment to the profession.

### 4.2. Motivational Determinants of High School Students’ Career Choices

We aimed to understand how and why students choose veterinary medicine as a career path. Those with stronger motivations tend to seek better job opportunities and are more willing to take risks in pursuit of their goals [23]. To enhance comparability, we applied the framework of Self-Determination Theory (SDT), and categorized the motivational characteristics and goals into two main types: intrinsic (e.g., interest in the field, interpersonal relationships, desire to help others, self-fulfilment, desire for independence, and hobbies [15,26,27]) and extrinsic (e.g., career prospects, prestige, social status, financial security, job stability, opinions of friends and family, and role models [14,26,27]). Our findings also confirmed gender-based differences in the importance of these motivational characteristics. For female students, intrinsic motivations related to their field of interest tend to play a more significant role in their career choice. As noted by Haase [27], intrinsic motivation is often a stronger predictor of career choice for females than academic performance (e.g., grades). In contrast, for males, this relationship may at times be reversed, with extrinsic characteristics playing a more prominent role.

### 4.3. Associations Between Age, Gender, Educational Background and Career Choices

Our results show that choosing veterinary medicine as a career is typically a very early decision, occurring even earlier than reported in other studies. Among Hungarian students, the average age of career commitment is approximately 10 years for females and 12 years for males. This aligns with the findings of Aschbacher, who observed that no students developed an interest in science after the 10th grade [29], and with other studies that also suggest early career choice [12,33].

In our sample, 22.0% of students attended specialized high schools, 71.7% attended general high schools, and only 5.4% studied in technical or vocational schools. Specialized schools were more commonly attended by female students and those from Budapest or its surroundings (Pest County), whereas technical schools were more frequently attended by male students and those from rural areas. One strategy to promote STEM careers is through the motivational design of science curricula in secondary education [28]. Students’ interest in these subjects plays a crucial role in shaping their career choices [34,35]. However, when science education fails to maintain students’ interest and motivation, those whose parents cannot afford private tutoring are likely to fall behind and may abandon their STEM career aspirations [29].

Career days and job fairs can significantly influence students’ engagement with specific professions [8,14]. To support this, UVMB organizes an annual Open Day, inviting high school students to visit the campus and explore the veterinary profession. The entire program is also available online to enhance accessibility. Although the online information about veterinary education is generally thorough and understandable, it is often not straightforward or practical enough to fully serve its purpose [1]. Additionally, few science teachers actively incorporate veterinary career topics into classroom discussions, despite their relevance to the curriculum [1]. This is a missed opportunity, as students whose teachers provide clear and comprehensive information about STEM careers exhibit greater intrinsic motivation towards these fields [28]. Our previous Hungarian study found that while job fairs and Open Days remain popular sources of information, most students prefer to gather details online [21].

### 4.4. Intrinsic Motivational Characteristics

Intrinsic motivational characteristics are among the first to consider when choosing a career path [28]. Altruism and “other orientation” are common motivating characteristics in both veterinary and human medicine [18,19,26,36,37,38]. The desire to help others and give back to the community is stronger in females, and this tendency often leads females interested in science, technology, engineering, and mathematics (STEM) to turn to medical career paths. However, Feakes’ et al. [18] did no find pronounced differences in altruism between genders in veterinary populations, which may be the result of the care-oriented nature of veterinary medicine. Students who express helping motivations related to science (e.g., “*I want to help people*”) are less likely to abandon their career goals [29]. In our study, female students scored slightly higher on the item “*I want to cure animals*” (4.41 vs. 3.95), and this finding is consistent with Holzer’s results, where female respondents showed significantly higher scores on similar altruistic intentions [26]. According to our results, 79.2% of our students stated that the desire to heal animals played a significant role in their decision to study veterinary medicine (scoring 4 or higher), in contrast to Cake’s study, where only 21% of veterinary students cited this motivation [12]. This might be one of the reasons why most Hungarian veterinary aspirants aim to pursue a career in small animal clinical practice.

We invited Hungarian high school students to rate their determination to become veterinarians using a Likert scale, based on the statement: “*I definitely want to become a veterinarian.*” A total of 89.7% of respondents rated their determination as 3 or higher, 66.8% as 4 or higher, and 51.6% rated it 5—indicating a strong, long-standing aspiration. However, the relevant international findings are inconsistent; Cake’s study reported that only 21% of students expressed this level of certainty [12], whereas Tomlin’s study found that 79.6% of students shared this sentiment [11]. Additionally, a notable proportion of the high school students (39.7% and 31.5%) reported that becoming a veterinarian feels more like a “calling” than just a career opportunity [26,39]. In most cases, “calling” refers to an individual’s deep, intrinsic sense of purpose or commitment to pursuing the profession, not merely as a job or career path, but as a meaningful life calling. This concept is often associated with a strong internal motivation to care for animals, contribute to animal and public health, and serve the community, regardless of financial reward or external recognition. In Hungary, this is a common narrative to shift the focus from difficult university exams, long working hours and self-sacrificing work conditions. On the one hand, it can give students and professionals the much-needed motivation to continue, but on the other hand, it may contribute to burnout as well. In our study, 77.6% of students agreed with the statement “*I can always learn something new*” (scoring 4 or higher), indicating that intellectual curiosity played a meaningful role in their motivation. In contrast, only 26% of students in Cake’s study identified this as a major characteristic [12]. As other studies showed, the medical or scientific orientation is also important: “*I am interested in science/healthcare*” was influential for a notable proportion of the students: 26.4–35.0% [12,26,39].

### 4.5. Extrinsic Motivational Characteristics

Extrinsic motivational characteristics are also important, since the lack of external incentives can steer students away from STEM careers and towards fields that offer more positive feedback [29]. Although male students scored slightly higher on extrinsic motivational characteristics (2.73 vs. 2.88), the pattern was not as distinct as in other studies. Only the items “*Easy to find a job*” (3.29 vs. 3.44) and “*Good salary*” (3.43 vs. 3.90) were rated as slightly more important by male students. In contrast, other international studies reported higher importance of extrinsic motivational characteristics such as income, social prestige, and career opportunities among male students [26]. Veterinary medicine is generally perceived as an important and respected profession, though typically less prestigious than human medicine. In our sample, 69.9% of the Hungarian respondents stated that this perception influenced their choice to pursue veterinary studies (scoring 4 or higher), whereas in Cake’s study, this factor played a considerably smaller role, influencing only 19% of respondents [12]. Salaries in the veterinary profession vary widely depending on specialization, location, practice size, and other variables. Although high earnings are possible in certain fields, this was a motivating factor for only 14.0% of students in Cake’s study [12] and 26.3% in Tomlin’s study [11]. Among Hungarian students, however, this motivation was far more prevalent: 52.3% rated salary as an important characteristic (score of 4 or higher). Given the social and economic development of Hungary and the broader region, higher education degrees such as this continue to be perceived as pathways to social and economic mobility.

Our findings show that students from rural areas seem to have a closer connection to the veterinary profession itself, as a higher proportion of these students stated that having veterinarians amongst their friends or family members was influential in their decision compared to students from urban areas. This is notable because individuals from families with a tradition of higher education are often influenced by those family members when making career choices [40,41]. While international studies indicate that only a small number of students had family or friends in agriculture [39] or veterinary medicine [10], those who did often found that this usually was the first step towards becoming a veterinarian, just as it usually ignites the first spark towards agricultural careers [14].

Interestingly, students from larger cities more frequently reported that they “*Always wanted to become a vet*” and placed somewhat greater importance on the profession’s perceived accessibility (“*Easy to find a job*”). However, students from the capital city rated “*Respected profession*”, “*Good salary*”, and “*Easy to find a job*” as considerably less important than any other sociodemographic group, even though they scored similarly high on “*Always wanted to become a vet*” as students from other urban areas. We interpreted this as part of the centralization of both intellectual and financial assets in the capital city, Budapest, which is prominent in most aspects of life in Hungary. These trends largely align with the findings reported by Villarroel [33].

### 4.6. Motivation Related to Animal Exposure

Veterinary medicine requires a certain approach and attachment to both animals and scientific studies. Those students who are seriously considering a future career working with animals are more likely to pursue one or more scientific subjects at A-level [8]. According to our results, 76.6% of the surveyed students indicated that their desire to work with animals significantly influenced their aspiration to become a veterinarian (scoring 4 or higher). This figure is slightly lower than that reported in Cake’s study (81.0%) [12], but higher than another study in which 71.6% of respondents expressed similar motivation [33]. In contrast, 94.6% of the Hungarian respondents indicated that they were strongly influenced by love of animals, a notably higher percentage. In our sample, more female students expressed a desire to work with animals compared to male students: 81.3% of females and 63% of males scored 4 or higher on the statement “*I want to work with animals.*” Robinson’s study suggests that younger students are more likely to consider careers involving animals, highlighting the importance of nurturing this interest early on [8].

Students from large cities were more likely to indicate that they would not consider working with animals. However, no statistically significant difference was found between students from urban and rural areas in this regard [8]. Our results show that students from centralized or larger cities are less likely to consider working with large animals—whether in clinical practice or state veterinary services. In contrast, the career choices of students from townships and small towns were more closely connected to animals and the land. These students scored higher on statements reflecting the influence on career choice such as “*I would like to work with animals*”, “*I nursed a sick animal back to health*”, and “*I am interested in agriculture*”, indicating a deeper personal connection with both animals and rural life.

### 4.7. Childhood Animal Exposure and Veterinary Field Choices

Our society is undergoing a shift, with an increasing number of students avoiding careers in livestock and food-chain-related veterinary fields. This development raises concerns about potential workforce shortages in these areas [26], a trend that is already becoming evident in Hungary [5]. The choice of veterinary field is influenced by multiple variables, including personal and financial reasons, as well as socio-economic background and upbringing, which are crucial in predicting a student’s choice of specific veterinary field [26]. In the UK, most students who pursued science at A-level had at least one or two pets during childhood, with some also having experience with horses [8]. Having a pet is quite common among veterinary aspirants. In our study, only 2.6% of our respondents reported growing up without any animals. Although exact figures vary across countries and over time, the proportion of students who had pets as children generally remains high, typically between 75.5% and 99.0% [12,26,33].

It appears that students who eventually pursue livestock practice often make this decision before starting university [42,43]. There is a significant association between growing up around livestock or gaining early farm experience and later choosing livestock or mixed animal practice. Similarly, experience with horses is frequently linked to choosing equine practice [13]. According to our results, the most influential characteristic in choosing farm animal practice is a student’s interest in agriculture. Notably, even among students with low motivation toward agriculture (score of 3 or lower), those who grew up with livestock were still more likely to express interest in farm animal practice. In other studies, while very few students had relatives in agriculture, those who did were significantly more likely to choose livestock or mixed animal practices (35.7% vs. 8.7% and 75% vs. 40%) [39]. These findings underscore the importance of engaging students with relevant backgrounds before the end of high school [43].

Serpell [13] reported that 35% of first-year veterinary students had notable experience on farms, whereas Robinson [8] noted that relatively few veterinary aspirants had lived or worked on a farm. Our findings are consistent with this trend: students from small towns or rural areas were more likely to choose livestock-oriented careers [3,33]. Specifically, students from villages and townships showed increased interest in farm animal practice (rising from 28% to 49.5%) and in state veterinary practice (from 5.4% to 11.9%).

Students who had only dogs or cats during childhood showed a strong preference for small animal practice (78.9%). These students were also open to careers in exotic animal practice (29.6%), laboratory work (22.5%), and education (21.1%), but demonstrated minimal interest in large animal fields: equine (9.9%), state veterinary work (2.8%), and farm animal practice (1.4%). While Serpell found this preference to be even more pronounced among students raised in urban environments, our study did not explicitly examine this characteristic [13]. In contrast, students who had horses as children showed a high preference for equine practice (83.1%), while also maintaining a strong interest in small animal medicine compared to the average (63.3% vs. 68.7%). These students also expressed slightly more interest in farm animal practice than the average (39.8% vs. 35.1%). This contrasts with Serpell’s findings, which suggested that students with horse experience tended to avoid both livestock and small animal practice, even if they had also owned cats or dogs [13]. Our findings show that the main motivational characteristics for mixed practice were very similar to those of equine and farm animal practice, suggesting the impression that the main predictor for pursuing a career as a mixed practice veterinarian was interest in large animal medicine, while interest in small animal medicine was secondary. Overall, these results emphasize the importance of early identification and recruitment of students with relevant motivations and backgrounds. Doing so allows for more effective resource allocation and supports the training of the most suitable future professionals for each veterinary field.

The patterns of one’s dominant motivational characteristics may relate to the students’ choice of career path and preferred field. Past experience is thought to strongly shape career intentions: for example, if a student has experience handling a certain species, it may give them the necessary confidence to pursue that specific path [44,45]. This might explain why our findings link interest in equine practice to experience with horses or why having experience with large animals might serve as a proxy for interest in food animal practice. Considering personality traits and drivers, such as emotional engagement with animals or business orientation, might help differentiate students who are not only interested in food animal medicine and veterinary public health, but are also well-suited for these fields [46].

### 4.8. Consideration of Alternative Career Paths

Veterinary medicine is generally perceived as a respected and important profession. However, some students report being discouraged from pursuing it, as it is not always regarded as a “proper” or equally prestigious career compared to human medicine or pharmacy [1]. With the profession increasingly shifting toward small animal medicine and high-end clinical care, a significant overlap has emerged between veterinary and human medicine. According to our findings, 40% of students interested in veterinary medicine also expressed interest in human medicine. Many students consider human medicine to carry greater prestige, a perception that some students attribute to the “poor marketing” of the veterinary profession [8]. Students who were undecided between veterinary and human medicine often expressed a greater affinity for animals but also faced significant pressure from family and peers to pursue human medicine instead [8]. In most years, there are new UVMB students who started their higher education journey at one of Hungary’s human medical universities due to external pressure and only later realized that a veterinary medical career might suit them better. These dynamics should be considered when recruiting future veterinary students. Human medicine is typically chosen at a later stage in life [47], suggesting the importance of maintaining students’ engagement with veterinary medicine throughout their academic journey. Without sustained encouragement and exposure, there is a risk of losing initially interested students to competing fields such as human medicine, which underscores the importance of extracurricular activities, summer camps, and experiential learning opportunities.

### 4.9. Experiential Learning and Veterinary Career Aspirations

Job shadowing refers to the practice in which a student spends time observing and assisting a professional in their daily work, offering firsthand insight into a given career. We strongly advocate for the benefits of job shadowing in veterinary medicine, as it allows students to gain a realistic understanding of the profession and helps clarify the path they might follow. For some, this may be discouraging; for others, it may further ignite their passion, often serving as a pivotal moment [8,10,20]. Having a strong role model might also lead a student to a specific field for various reasons. This may be due to the interest sparked, or the confidence gained, while observing and assisting in that field [44,45]. In addition, the external pressure of meeting a mentor’s expectations, even if only subconsciously, may also play a role.

Among our respondents, 53% reported some form of job shadowing experience. This practice is especially prevalent among highly motivated students. Job shadowing, nursing animals back to health, and having veterinarian acquaintances were all found to be strongly interrelated. Even routine veterinary visits can be influential: in one study, 40.5% of veterinary students cited such visits as a decisive moment in their career choice [11], whereas for some high school students, the same experience was rather discouraging [8]. In our study, students who were less influenced by the experience of nursing a sick animal back to health but who had horses were still likely to have engaged in job shadowing. This is likely due to closer contact with their horse’s veterinarian, who could be consulted more easily. These insights highlight the importance of creating and promoting job shadowing opportunities—particularly for students who lack personal connections to veterinary professionals, as our results suggest that they may have more difficulty finding welcoming mentors.

Extracurricular programs might also offer valuable opportunities to spark interest in STEM careers, but only if they successfully present science in a relatable and engaging way. If not, they may inadvertently discourage students [29]. Creative, arts-based, and informal, outdoor, experience-driven science programs have been shown to ignite interest in science even among those with no prior connection to it [48]. Moreover, these programs have the potential to enhance academic performance through the development of stronger intrinsic motivation [49].

In the United States, organizations such as 4-H and FFA offer programs that promote agricultural careers among high school students and young adults. According to data from the Michigan State University College of Veterinary Medicine and other sources, their competitions and job fairs have successfully guided many students toward veterinary medicine [10,14]. Hungarian veterinary students also organize an annual competition for high school students, where participants learn extensively about animals and the veterinary profession. In our study, 45.8% of students expressed interest in participating in such a competition, while 90.4% stated they would gladly attend a veterinary-themed summer camp if one were available. Although UVMB does not currently organize such camps, the university actively supports similar initiatives.

### 4.10. Limitations

It is important to note that the findings of the present study are based on voluntary participation and convenience sampling, which may have introduced selection bias and reduced the representativeness of the sample. Consequently, the findings should not be generalized to the broader student population. Another limitation is that the questionnaire instrument was not fully validated: while some items were adapted from existing Self-Determination Theory (SDT) measures, others were developed specifically for this study without prior psychometric testing. This raises potential concerns regarding the reliability and construct validity of certain scales. Finally, as with many cross-sectional survey designs, the results reflect associations rather than causal relationships. Therefore, this study should be viewed as exploratory in nature, aiming to highlight indicative trends and associations that warrant further investigation.

## 5. Conclusions

To the best of our knowledge, this study represents the first large-scale scientific investigation into the key motivational characteristics of Hungarian high school students interested in veterinary medicine. Most existing research on this topic has been conducted in other regions, with the most comprehensive studies originating from the United States and the United Kingdom. These countries have markedly different historical, social and economic contexts, as well as educational systems, compared to those of Central and Eastern European countries. Therefore, the results of the current study may add new perspectives to this topic and can potentially be extrapolated to other countries in the region. By analyzing the socio-demographic background of respondents, we identified the primary motivational characteristics driving students to pursue veterinary medicine. This knowledge can help in the development of supportive preparatory programs that better equip students to meet university admission requirements. Moreover, during the enrolment process, it becomes possible to identify and reward applicants who demonstrate strong motivation and a natural fit for the profession, thereby increasing their chances of success, not only in gaining admission, but also in completing their studies and thriving in their future careers. Our findings can serve as a basis for designing and justifying future targeted programs, developed in collaboration with universities. The aim of these initiatives will be to help students assess their own abilities, gain early insight into various career options, and make more informed career choices, as no such programs are currently available for Hungarian high school students.

## Figures and Tables

**Table 1:** Respondents’ Age and Educational Background by Gender and Place of Residence (*N* = 428).

Socio-Demographic Characteristics	Females	Males	Capital	County Seat	City	Township/Village
*N*	317	111	88	82	157	101
Gender						
Females	-	-	80.7%	72.0%	74.5%	69.3%
Males	-	-	19.3%	28.0%	25.5%	30.7%
Age						
Mean	17.6	18.2	17.5	18.1	17.7	17.8
SD	±2.5	±5.3	±3.1	±3.4	±3.7	±3.5
Age of career decision (years)	10.9	12.8	12.0	11.6	11.3	10.8
Secondary education						
Specialized high school	22.7%	19.8%	31.8%	26.8%	17.8%	15.8%
General high school	72.2%	70.3%	64.8%	68.3%	79.6%	68.3%
Technical school	3.8%	9.9%	2.3%	1.2%	2.5%	15.8%

**Table 2:** Descriptive statistics of motivational characteristics by gender (*N* = 428).

Motivational Characteristics	Females			Males		
	Mean	SD	IQR	Mean	SD	IQR
Friends/family is a vet	2.11	1.38	2	2.07	1.15	2
Interested in agriculture	2.73	1.33	2	2.88	1.43	2
Nursing a sick animal	3.30	1.43	3	2.79	1.27	2
Easy to find a job	3.29	1.22	2	3.44	1.35	3
Good salary	3.43	1.17	1	3.90	1.18	2
Respected profession	3.98	1.17	1	3.96	1.23	2
Always wanted to become a vet	3.63	1.32	2	3.24	1.23	2
Universal knowledge	4.05	1.18	2	3.59	1.32	2
Learning new things	4.35	0.98	1	3.86	1.17	2
I want to work with animals	4.41	1.02	1	3.95	1.21	2
I want to cure animals	4.40	0.95	1	3.86	1.04	2
I like animals	4.67	0.85	0	4.34	1.03	1

Note: Likert scale from 1 to 5, where 1 means “Not at all” and 5 means “Very strongly”.

**Table 3:** Preferred Professional Fields by Gender and Childhood Animal Exposure (*N* = 428).

Preferred Professional Fields	Gender		Childhood Animal Exposure (Species Present in the Household or Experienced)			
	Females	Males	Dogs/Cats	Horses	Livestock	Exotic Animals
Small animal practice	72.6%	57.7%	78.9%	63.3%	63.0%	74.1%
Equine practice	53.0%	20.7%	9.9%	83.1%	53.3%	46.3%
Exotic animal practice	34.4%	28.8%	29.6%	24.1%	23.0%	50.2%
Laboratory work	23.7%	23.4%	22.5%	14.5%	21.8%	28.9%
Farm animal practice	32.8%	41.4%	1.4%	39.8%	63.6%	39.3%
Academic career	12.6%	13.5%	21.1%	7.2%	8.5%	15.4%
Veterinary public health	8.2%	8.1%	2.8%	8.4%	13.3%	9.5%

Note: More than one option could be selected.

## Data Availability

The original contributions presented in this study are included in the article/Supplementary Material. Further inquiries can be directed to the corresponding author.

## Supplementary Materials

The following supporting information can be downloaded at: https://www.mdpi.com/article/10.3390/vetsci12121189/s1, Supplementary Questionnaire, CART_for_Szucs_et_al_2025.
