# Supplementary material for: Predicting Veterinary Career Intentions Using Motivational Characteristics: A Survey Study Among Hungarian Students
Source: Vet Sci. 2025 Dec 12;12(12):1189. doi: 10.3390/vetsci12121189 (PMC12737562; doi:10.3390/vetsci12121189)
Supplement: Supplementary file 1 [file vetsci-12-01189-s001.zip › vetsci-4022081-Supplementary Material.pdf]

Supplementary Material

Questionnaire on Career Orientation

During the following questionnaire, you will find questions about your motivations for becoming a veterinarian. This questionnaire is part of a research project aiming at understanding the most important motivating factors for veterinary aspirants. The questionnaire is anonymous and takes only 5–10 minutes to complete. Your responses are important and will help us develop new programs and opportunities tailored to your needs.

Section 1

1. Your gender
    a. Female
    b. Male
2. Your age (years):__________
3. Type of school you attend
    a. Specialized high school
    b. General high school
    c. Technical school
    d. Other:____________________
4. How would you rate the biology education in your school on a scale from 1 to 5? (1: very poor, 5: excellent)

    1. 2. 3. 4. 5.
5. How would you rate the chemistry education in your school on a scale from 1 to 5? (1: very poor, 5: excellent)

    1. 2. 3. 4. 5.
6. Which county do you live in?
    a. Bács-Kiskun county
    b. Baranya county
    c. Békés county
    d. Borsod-Abaúj-Zemplén county
    e. Csongrád-Csanád county
    f. Fejér county
    g. Győr-Moson-Sopron county

h. Hajdú-Bihar county
i. Heves county
j. Jász-Nagykun-Szolnok county
k. Komárom-Esztergom county
l. Nógrád county
m. Pest county
n. Somogy county
o. Szabolcs-Szatmár-Bereg county
p. Tolna county
q. Vas county
r. Veszprém county
s. Zala county
t. Budapest (capital)

7. What type of settlement do you live in?
   a. Capital
   b. County seats
   c. City
   d. Township/Village

Section 2

1. How true is the following statement for you on a scale from 1 to 5? (1: I do not agree at all, 5: I completely agree)

   "I definitely want to become a veterinarian."

   1. 2. 3. 4. 5.

2. How old were you when you decided you want to become a veterinarian?

   At the age of __________years

3. What other careers would you consider beside veterinary medicine?

(You may choose more than one option)

   a. I am not considering any other careers
   b. Medical doctor
   c. Dentist
   d. Nurse
   e. Paramedic
   f. Agricultural engineer

g. Animal husbandry engineer
h. Biologist
i. Zoologist
j. Veterinary nurse
k. Other:________________________

4. What kind of animals did you spend a lot of time with as a child?
(You may choose more than one option)
   a) We did not have any animals
   b) Dog
   c) Cat
   d) Hamster/Guinea pig/Rabbit
   e) Bird
   f) Fish
   g) Reptile/Amphibian
   h) Horse
   i) Swine
   j) Cattle
   k) Sheep/Goat
   l) Poultry
   m) Other:________________________

5. Which areas of veterinary medicine are you interested in?
(You may choose more than one option)
   a) Treating dogs and cats
   b) Treating livestock (cattle, swine, poultry)
   c) Treating horses
   d) Treating exotic animals (fish, reptiles, birds, small mammals, etc.)
   e) Laboratory/Research
   f) Teaching
   g) Public / Official veterinary practice
   h) Other:____________________________

6. How much did the following factors influence your decision to become a veterinarian on a scale from 1 to 5? (1: Not at all, 5: Very strongly)

a) I would like to work with animals	1	2	3	4	5
b) I would like to heal animals	1	2	3	4	5

c)	I have always wanted to become a vet	1	2	3	4	5
d)	I like animals	1	2	3	4	5
e)	I am interested in agriculture	1	2	3	4	5
f)	A family member or friend is a vet	1	2	3	4	5
g)	I nursed a sick animal back to health	1	2	3	4	5
h)	It is easy to find a job	1	2	3	4	5
i)	It is a respected profession	1	2	3	4	5
j)	Good salary	1	2	3	4	5
k)	I can always learn something new	1	2	3	4	5
l)	Veterinarians are needed all over the word	1	2	3	4	5
m)	Other ______________________________	1	2	3	4	5

7. Have you ever spent time with a veterinarian to get to know the profession better?
   a) Yes
   b) No
8. Are you interested in the Jozsef Marek Scholarship?
   a) Yes
   b) No
9. Have you heard about the Animal Health Competition?
   a) Yes
   b) No
10. Would you like to participate in programmes (e.g., a summer camp) that introduce you to the veterinary profession?
    a) Yes
    b) No
11. Would a free online preparatory course help you prepare for the advanced level Matura exams?
    a) Yes
    b) No
12. Have you heard of the biology and chemistry entrance exam preparatory course offered by UVMB?
    a) Yes
    b) No
